# Case-ascertainment of acute myocardial infarction hospitalizations in cancer patients: a cohort study using English linked electronic health data

**DOI:** 10.1093/ehjqcco/qcab045

**Published:** 2021-06-22

**Authors:** Briana Coles, Lucy Teece, Clive Weston, Mark A de Belder, Clare Oliver-Williams, Catherine A Welch, Mark J Rutherford, Paul C Lambert, Patrick Bidulka, Lizz Paley, Dorothea Nitsch, John Deanfield, Mick D Peake, David Adlam, Michael J Sweeting

**Affiliations:** Biostatistics Research Group, Department of Health Sciences, University of Leicester, Leicester, UK; National Cancer Registration and Analysis Service, Public Health England, London, UK; Biostatistics Research Group, Department of Health Sciences, University of Leicester, Leicester, UK; National Cancer Registration and Analysis Service, Public Health England, London, UK; Department of Cardiology, Glangwili General Hospital, Carmarthen, UK; National Institute for Cardiovascular Outcomes Research (NICOR), Barts Health NHS Trust, London, UK; Biostatistics Research Group, Department of Health Sciences, University of Leicester, Leicester, UK; National Cancer Registration and Analysis Service, Public Health England, London, UK; Biostatistics Research Group, Department of Health Sciences, University of Leicester, Leicester, UK; National Cancer Registration and Analysis Service, Public Health England, London, UK; Biostatistics Research Group, Department of Health Sciences, University of Leicester, Leicester, UK; Biostatistics Research Group, Department of Health Sciences, University of Leicester, Leicester, UK; Department of Medical Epidemiology and Biostatistics, Karolinska Institutet, Stockholm, Sweden; Department of Non-Communicable Disease Epidemiology, London School of Hygiene & Tropical Medicine, London, UK; National Cancer Registration and Analysis Service, Public Health England, London, UK; Department of Non-Communicable Disease Epidemiology, London School of Hygiene & Tropical Medicine, London, UK; National Institute for Cardiovascular Outcomes Research (NICOR), Barts Health NHS Trust, London, UK; Department of Clinical Science, Institute of Cardiovascular Science, University College London, London, UK; National Cancer Registration and Analysis Service, Public Health England, London, UK; Department of Respiratory Medicine, University of Leicester, Leicester, UK; Department of Cardiovascular Sciences and NIHR Leicester Biomedical Research Centre, University of Leicester, Leicester, UK; Biostatistics Research Group, Department of Health Sciences, University of Leicester, Leicester, UK; National Cancer Registration and Analysis Service, Public Health England, London, UK

**Keywords:** Cardio-oncology, Myocardial infarction, Cancer, Case-ascertainment, MINAP, HES

## Abstract

**Aims:**

To assess the recording and accuracy of acute myocardial infarction (AMI) hospital admissions between two electronic health record databases within an English cancer population over time and understand the factors that affect case-ascertainment.

**Methods and results:**

We identified 112 502 hospital admissions for AMI in England 2010–2017 from the Myocardial Ischaemia National Audit Project (MINAP) disease registry and hospital episode statistics (HES) for 95 509 patients with a previous cancer diagnosis up to 15 years prior to admission. Cancer diagnoses were identified from the National Cancer Registration Dataset (NCRD). We calculated the percentage of AMI admissions captured by each source and examined patient characteristics associated with source of ascertainment. Survival analysis assessed whether differences in survival between case-ascertainment sources could be explained by patient characteristics. A total of 57 265 (50.9%) AMI admissions in patients with a prior diagnosis of cancer were captured in both MINAP and HES. Patients captured in both sources were younger, more likely to have ST-segment elevation myocardial infarction and had better prognosis, with lower mortality rates up to 9 years after AMI admission compared with patients captured in only one source. The percentage of admissions captured in both data sources improved over time. Cancer characteristics (site, stage, and grade) had little effect on how AMI was captured.

**Conclusion:**

MINAP and HES define different populations of patients with AMI. However, cancer characteristics do not substantially impact on case-ascertainment. These findings support a strategy of using multiple linked data sources for observational cardio-oncological research into AMI.

## Introduction

Multimorbid patients make up the majority of hospital admissions and account for significant secondary care healthcare costs but are frequently excluded from clinical trials.^1–3^ For example, clinical trials on hospital management of acute myocardial infarction (AMI) may exclude cancer patients. This is of particular concern because cardiovascular issues, including cardiotoxicity, are common in cancer patients.^4^ The identification of AMI outcomes using routinely collected electronic health record databases is a promising approach that provides a scalable means for large ‘big data’ studies that limits both research costs and patient inconvenience.^5^ However, the recording of AMI in databases amongst patients with a previous history of cancer has not been explored.

The Myocardial Ischaemia National Audit Project (MINAP) is a large clinical audit with detailed, patient-level data on patients admitted to hospitals in England, Wales, and Northern Ireland with AMI since 2000.^6^^,^^7^ It is apparent however that some AMI hospitalizations are not captured by the registry. Previous researchers studying MINAP records prior to 2009, found that only around half of patients with at least one record of non-fatal AMI were captured in MINAP when compared with AMI capture in hospital episode statistics (HES) data (routinely collected secondary care data coded by non-clinical coding clerks), and primary care data from the Clinical Practice Research Datalink (CPRD).^8^ This may partly be due to incomplete hospital-level case-ascertainment but also likely arises because MINAP is targeted primarily to capture AMI caused by atherothrombotic coronary artery disease or Type I AMI.^9^

Because MINAP is an audit predominantly recorded by cardiology services, patients whose AMI care is primarily supported by another specialty (e.g. cancer or palliative care) may not be referred to cardiology services and as a result not be captured by MINAP. This may be more likely for patients with more advanced disease. On the other hand, HES is a clinical coding database and ascertainment of AMI, particularly through recording in the first diagnostic position, could be impacted by the presence of another dominant disease at the time of coding (e.g. cancer). This might be particularly the case for more advanced cancer (higher stage and grade).

In this article, we sought to investigate ascertainment of hospitalized AMI in an English cancer population utilizing data from the Virtual Cardio-Oncology Research Initiative (VICORI), which is a research platform that links existing national English cancer registry and cardiovascular audit data through a unique identifier.^10^ Through linkage of the National Cancer Registration Dataset (NCRD)^11^ with both MINAP and HES we investigate firstly, whether individual or multiple data resources are required for the ascertainment of AMI in cancer patients, secondly whether cancer characteristics (site, stage, and grade) impact on ascertainment of AMI and thirdly to investigate the differences in characteristics and survival of cancer patients with AMI captured by MINAP, HES, or both data sources.

## Methods

### Study population

AMI hospital admissions were identified from English MINAP and/or HES Admitted Patient Care (APC) datasets. The study population included all AMI admissions to hospitals in England between 1 January 2010 and 31 December 2017 in patients aged ≥40 who had a previous diagnosis of cancer (up to 15 years prior to the AMI admission) recorded in the NCRD held by the National Cancer Registration and Analysis Service (NCRAS).^11^ Cancer diagnoses were identified using ICD-10 codes C00-D48. A patient could have more than one AMI admission included in the study if they occurred on different days.

Linkage between NCRD and HES APC was performed using matching criteria based on NHS number, date of birth, sex and postcode, with any matches ranked 1–5 included (see Supplementary material online, *Appendix A*). Linkage between NCRD and MINAP meanwhile required an exact match by both NHS number and date of birth.

AMI hospital admissions in MINAP were identified based on discharge diagnosis, cardiovascular biomarkers, and electrocardiographic findings^8^^,^^9^ (Supplementary material online, *Appendix B*). MINAP contains detailed diagnostic information from electrocardiograms and cardiac biomarkers, not available in other routinely collected datasets, which allows accurate phenotyping of acute myocardial infarction (AMI) into ST-segment elevation myocardial infarction (STEMI) and non-ST-segment elevation myocardial infarction (NSTEMI). Only MINAP admissions that could be classified as STEMI or NSTEMI were included in our study. AMI admissions in HES were identified by ICD-10 codes I21–I23 as the primary diagnosis for the first episode (continuous period of care under one consultant) of a spell (admission). In HES, STEMI and NSTEMI admissions were identified using ICD-10 codes (Supplementary material online, *Appendix C*). Admissions captured in both MINAP and HES were assigned the phenotype indicated in MINAP. We performed a sensitivity analysis to assess the influence on case-ascertainment results of AMI codes in secondary diagnosis positions or in subsequent episodes.

Follow-up for mortality was through linkage with the Office of National Statistics (ONS) Mortality Registry. The last date of follow-up for all patients was 31 December 2018.

### Matching AMI admissions from MINAP and HES

AMI admissions were considered to represent a match or part of the same acute episode if the admission date in MINAP corresponded to an AMI admission in HES within 30 days. MINAP admissions were considered the gold standard, therefore, if multiple admissions in HES were within 30 days of a MINAP admission date the HES admission with the closest date was selected as the single matched event, to prevent double counting. MINAP admissions without a HES record within 30 days were classified as ‘MINAP only’ and HES admissions without a MINAP admission within 30 days were classified as ‘HES only’. For matched events, the MINAP admission date was used. We performed a sensitivity analysis to determine case-ascertainment if the match window between HES and MINAP admissions was increased to 60 days, 90 days, and no time restriction.

### Covariates

Charlson comorbidity score was calculated from comorbidities in inpatient diagnostic HES fields within 1 month–15 years before the first AMI admission using previously defined coding algorithms.^12^^,^^13^ Index of multiple deprivation income quintiles, a relative measure of deprivation at small area level, was assigned based on the postcode of residence closest to the cancer diagnosis date. Ethnicity was categorized as White, Mixed, Asian, Black, other, or unknown. Age at time of AMI admission was categorized as 40–59, 60–69, 70–79, 80–89, 90+ years. Geographical region reflects the English region based on postcode of residence when the cancer was diagnosed, categorized as London, Midlands and East, North, South. Other cancer characteristics were obtained from the NCRD.

### Statistical analysis

Patient characteristics for first AMI admission were reported for all patients and stratified by case-ascertainment source. Pearson’s Chi-squared tests (categorical) and Kruskal–Wallis tests (continuous) were performed to examine the association between patient characteristics and case-ascertainment source for first admissions. The percentage of AMI admissions captured by each source were displayed in stacked bar charts.

Annual ICD-10 coding trends for AMI admissions in HES were examined over time. Furthermore, we examined how ICD-10 codes for HES admissions correlated with case-ascertainment in MINAP. For AMI admissions captured in MINAP only we investigated whether a HES admission with a non-myocardial infarction (MI) primary diagnosis occurred within 30 days. The non-MI primary diagnosis ICD-10 chapters were reported, and a more detailed examination was performed for chapters that commonly featured in the admission data.

Survival analyses were performed based on the time from the first AMI admission for each patient. Kaplan–Meier survival estimates were presented for all patients, and separately by first AMI phenotype. Flexible parametric survival models were implemented to assess if differences in survival between case-ascertainment sources could be explained by patient characteristics. A restricted cubic spline was used to model the baseline cumulative hazard of mortality, with 4 degrees of freedom selected based on Akaike’s information criterion. Case-ascertainment source was included in the model as a three-level categorical covariate interacted with time to allow for non-proportional hazards between the levels. This was achieved by including two additional restricted cubic splines for levels 2 and 3 of the covariate. All other covariates included in the model were considered as main effects only. For the survival analysis, Mixed, Asian, Black, and Other ethnic groups were combined into non-White. Standardized survival curves were obtained by case-ascertainment source, standardizing across the covariate distribution for all patients. Analyses included the following covariates: age group; sex; ethnicity; comorbidity score; AMI phenotype; year of AMI admission; deprivation; geographical region; years between cancer diagnosis and AMI admission; number of previous tumours; and cancer subtype categorized as invasive or non-invasive/non-melanoma skin cancer. Analyses were repeated stratified by AMI phenotype, where standardization was across the covariate distribution for patients in the specified strata. All analyses were conducted on records with no missing data in the relevant covariates (a complete case analysis).

## Results

**Figure 1:**
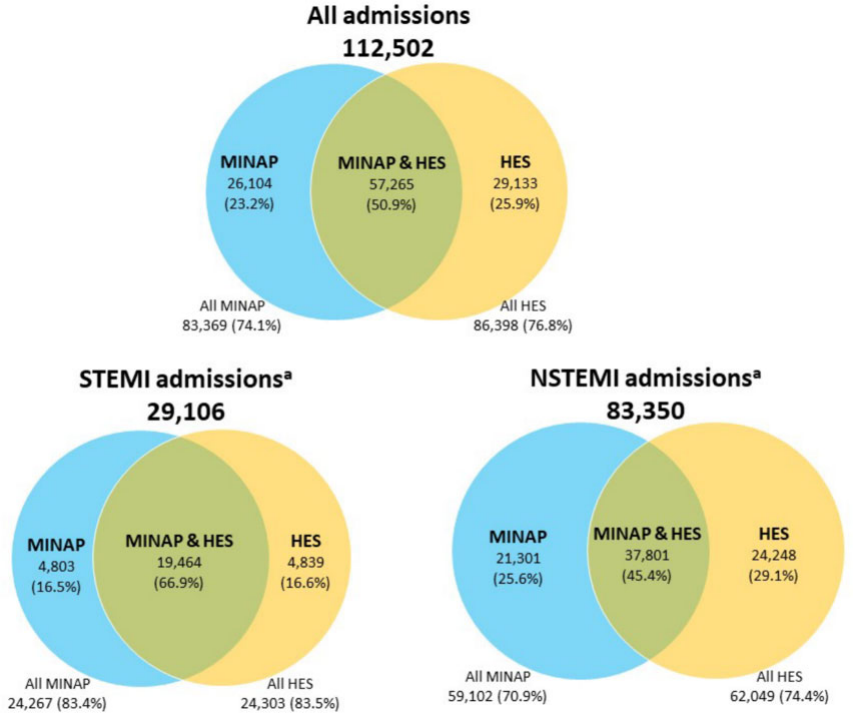
Case-ascertainment of AMI hospitalizations across disease registry (MINAP) and hospital care (HES) sources for a population of cardio-oncology patients in total and by AMI phenotype (STEMI and NSTEMI). ^a^Forty-six HES admissions were of unknown phenotype.

Between 2010 and 2017, we identified 112 502 hospital admissions for AMI across MINAP and HES for 95 509 patients who had had a previous cancer diagnosis up to 15 years prior to the admission. There was an average of nearly 4 years between cancer diagnosis and first AMI admission, with 8364 (7.4%) of cancer diagnoses occurring within 3 months prior to the AMI admission. Just over half of all AMI admissions were captured in both MINAP and HES, 57 265 (50.9%), with a further 26 104 (23.2%) ascertained only in MINAP and 29 133 (25.9%) ascertained only in HES (*Figure 1*).

### Factors affecting case-ascertainment

Compared with patients whose first AMI admission was captured only in MINAP, HES only patients had a higher median age at admission (1.8 years older); were more likely to be female, have NSTEMI, reside in London, Midlands and East, or South; and had worse 30-day survival (*P* < 0.001 for all, *Table 1*). Patients captured in both MINAP and HES had a longer median time between cancer diagnosis and AMI admission and fewer primary tumours. Other cancer characteristics were similar between all three case-ascertainment sources.

**Table 1:** Patient characteristics by case-ascertainment source based on first AMI admission

	Case-ascertainment source			Total
	MINAP and HES	MINAP only	HES only	
Number of patients	48 569	22 571	24 369	95 509
Age	78.0 (69.9–84.5)	79.5 (72.0–85.6)	81.3 (73.4–87.4)	79.2 (71.2–85.6)
Age category				
40–59	3711 (7.6%)	1230 (5.4%)	1159 (4.8%)	6100 (6.4%)
60–69	8518 (17.5%)	3308 (14.7%)	3030 (12.4%)	14 856 (15.6%)
70–79	15 752 (32.4%)	7221 (32.0%)	6771 (27.8%)	29 744 (31.1%)
80–89	16 362 (33.7%)	8467 (37.5%)	9636 (39.5%)	34 465 (36.1%)
90+	4226 (8.7%)	2345 (10.4%)	3773 (15.5%)	10 344 (10.8%)
Sex				
Male	31 937 (65.8%)	14 511 (64.3%)	14 775 (60.6%)	61 223 (64.1%)
Female	16 632 (34.2%)	8060 (35.7%)	9594 (39.4%)	34 286 (35.9%)
Ethnicity				
White	35 341 (96.6%)	16 325 (95.9%)	17 756 (95.8%)	69 422 (96.3%)
Mixed	60 (0.2%)	36 (0.2%)	33 (0.2%)	129 (0.2%)
Asian	725 (2.0%)	374 (2.2%)	424 (2.3%)	1523 (2.1%)
Black	232 (0.6%)	164 (1.0%)	184 (1.0%)	580 (0.8%)
Other	225 (0.6%)	117 (0.7%)	130 (0.7%)	472 (0.7%)
MI phenotype				
NSTEMI	30 896 (63.6%)	18 150 (80.4%)	20 045 (82.4%)	69 091 (72.4%)
STEMI	17 673 (36.4%)	4421 (19.6%)	4293 (17.6%)	26 387 (27.6%)
Charlson comorbidity score				
0	9287 (19.1%)	3380 (15.0%)	4352 (17.9%)	17 019 (17.8%)
1	4776 (9.8%)	2052 (9.1%)	2081 (8.5%)	8909 (9.3%)
2	13 801 (28.4%)	5584 (24.7%)	6492 (26.6%)	25 877 (27.1%)
3	8334 (17.2%)	4087 (18.1%)	4146 (17.0%)	16 567 (17.3%)
4+	12 367 (25.5%)	7463 (33.1%)	7298 (29.9%)	27 128 (28.4%)
Index of multiple deprivation quintile				
1—least deprived	9812 (20.2%)	4426 (19.6%)	4933 (20.2%)	19 171 (20.1%)
2	10 813 (22.3%)	4895 (21.7%)	5352 (22.0%)	21 060 (22.1%)
3	10 383 (21.4%)	4887 (21.7%)	5209 (21.4%)	20 479 (21.4%)
4	9224 (19.0%)	4418 (19.6%)	4731 (19.4%)	18 373 (19.2%)
5—most deprived	8337 (17.2%)	3945 (17.5%)	4144 (17.0%)	16 426 (17.2%)
Geographical region				
London	3128 (6.4%)	2082 (9.2%)	2636 (10.8%)	7846 (8.2%)
Midlands and East	14 471 (29.8%)	6636 (29.4%)	8089 (33.2%)	29 196 (30.6%)
North	17 328 (35.7%)	7654 (33.9%)	6591 (27.0%)	31 573 (33.1%)
South	13 642 (28.1%)	6199 (27.5%)	7053 (28.9%)	26 894 (28.2%)
30-day mortality	4926 (10.1%)	3381 (15.0%)	5456 (22.4%)	13 763 (14.4%)
Cancer characteristics				
Years since most recent cancer diagnosis	4.0 (1.5–7.7)	3.6 (1.2–7.5)	3.8 (1.3–7.8)	3.9 (1.4–7.7)
Number of previous tumours				
1	41 516 (85.5%)	19 102 (84.6%)	20 583 (84.5%)	81 201 (85.0%)
2	5957 (12.3%)	2908 (12.9%)	3109 (12.8%)	11 974 (12.5%)
3	927 (1.9%)	456 (2.0%)	563 (2.3%)	1946 (2.0%)
4+	169 (0.3%)	105 (0.5%)	114 (0.5%)	388 (0.4%)
Tumour stage				
0	4411 (28.4%)	2020 (28.1%)	2165 (28.6%)	8596 (28.4%)
1	4141 (26.6%)	1777 (24.7%)	1809 (23.9%)	7727 (25.5%)
2	3147 (20.2%)	1501 (20.9%)	1435 (18.9%)	6083 (20.1%)
3	2067 (13.3%)	968 (13.5%)	986 (13.0%)	4021 (13.3%)
4	1783 (11.5%)	923 (12.8%)	1179 (15.6%)	3885 (12.8%)
Tumour grade				
G1	3779 (18.8%)	1857 (18.9%)	1848 (19.2%)	7484 (18.9%)
G2	10 096 (50.1%)	4970 (50.6%)	4731 (49.2%)	19 797 (50.0%)
G3	5741 (28.5%)	2755 (28.1%)	2800 (29.1%)	11 296 (28.6%)
G4-6	519 (2.6%)	233 (2.4%)	230 (2.4%)	982 (2.5%)
Tumour site				
Breast	3317 (6.8%)	1560 (6.9%)	1833 (7.5%)	6710 (7.0%)
Bronchus, lung	1829 (3.8%)	1008 (4.5%)	1157 (4.7%)	3994 (4.2%)
Colorectal	3595 (7.4%)	1970 (8.7%)	1843 (7.6%)	7408 (7.8%)
Melanoma and other skin	17 088 (35.2%)	7207 (31.9%)	8233 (33.8%)	32 528 (34.1%)
Other	15 594 (32.1%)	7616 (33.7%)	8006 (32.9%)	31 216 (32.7%)
Prostate	7146 (14.7%)	3210 (14.2%)	3297 (13.5%)	13 653 (14.3%)

Data are presented as median (IQR) for continuous measures, and *n* (%) for categorical measures. Among all patients the following were missing: ethnicity *n* = 23 383 (24.5%), AMI phenotype *n* = 31 (0.0%), Comorbidity index *n* = 9 (0.00%), tumour stage *n* = 65 197 (68.3%), tumour grade *n* = 55 950 (58.6%). Note, demographic data are provided for the first AMI admission only. Subsequent admissions were ignored. Cancer characteristics are reported for most recent (previous) cancer diagnosis. Index of multiple deprivation was recorded in NCRAS at date of most recent cancer diagnosis, which may be up to 15 years prior to AMI admission.

**Figure 2:**
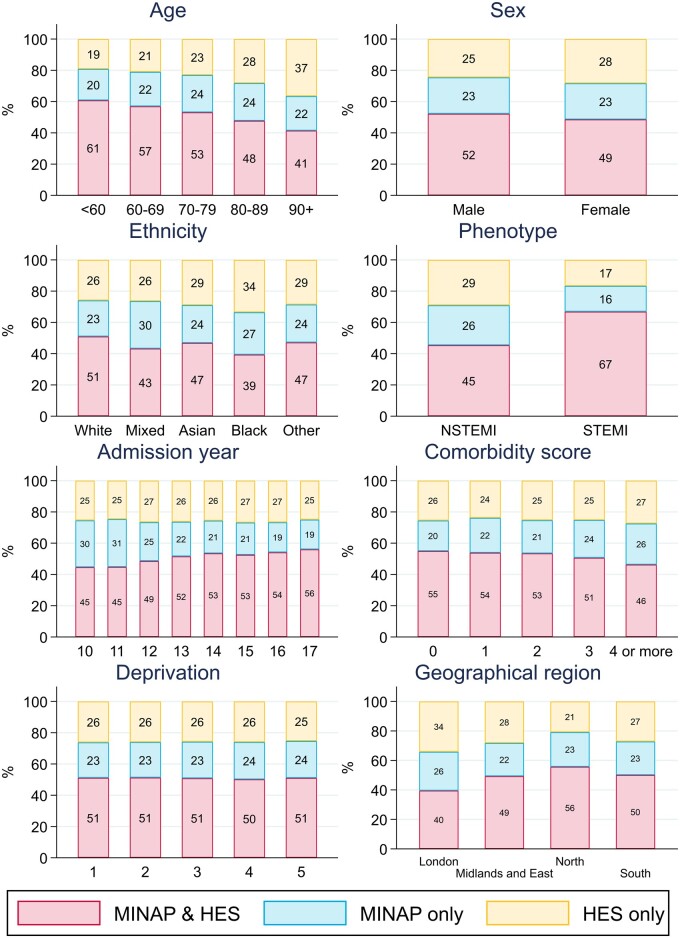
Case-ascertainment of *N* = 112 502 AMI hospitalizations across disease registry (MINAP) and hospital care (HES) sources for a population of cardio-oncology patients, by sex, AMI phenotype, age, calendar year, ethnicity, and deprivation quintile. Admission year includes 2010–2017. Ethnicity was unknown for 27 394 (24.3%) admissions. AMI phenotype was unknown for 46 (<0.1%) admissions. Comorbidity score was unknown for 10 (<0.1%) admissions. Deprivation quintile: 1 = least deprived; 5 = most deprived.

**Figure 3:**
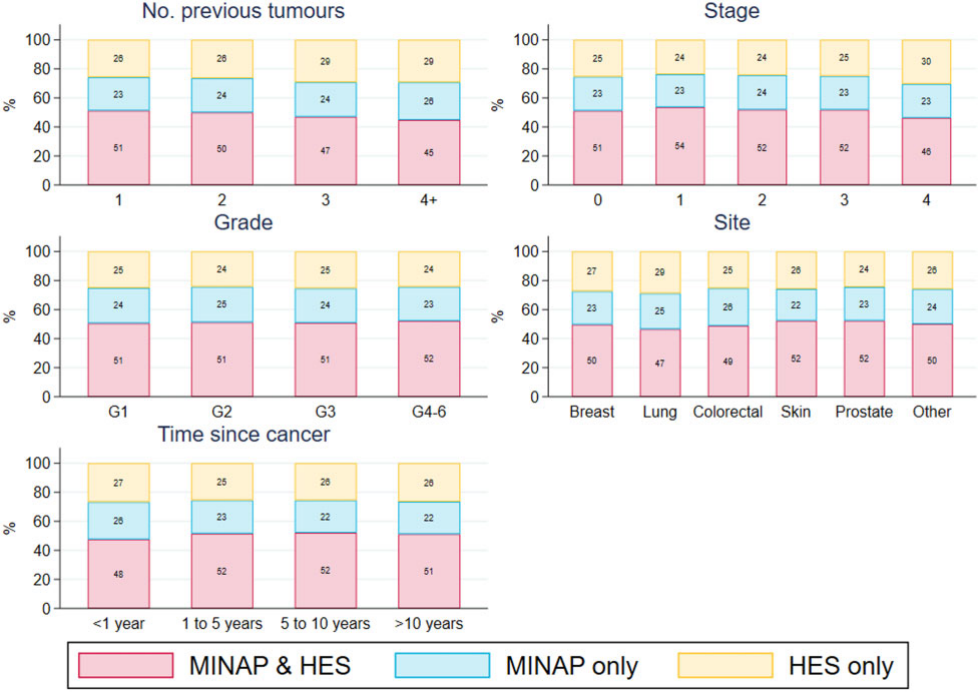
Case-ascertainment of *N* = 112 502 AMI hospitalizations across disease registry (MINAP) and hospital care (HES) sources for a population of cardio-oncology patients, by number of previous tumours, cancer stage, cancer grade, and cancer site. Stage was missing for 77 077 (68.5%) admissions. Grade was missing for 65 924 (58.6%) admissions.

Case-ascertainment in MINAP decreased markedly for patients aged 80 or above with MINAP failing to capture over a quarter of octogenarians and over a third of nonagenarians (*Figure 2*, Supplementary material online, *Table*  *S1*). Only 62% of admissions for nonagenarians with NSTEMI were captured in MINAP (Supplementary material online, *Figure*  *S1*). MINAP captured 84% of STEMI hospital admissions but only 71% of NSTEMI admissions in the cohort. The percentage of AMI admissions captured in both data sources improved over time rising from 45% of admissions captured in 2010 to 56% in 2017, whilst the percentage of cases captured in HES only remained stable. This improvement in ascertainment was most evident for STEMI admissions (Supplementary material online, *Figure*  *S1*). Patients with multiple comorbidities were less likely to be captured in MINAP (*Figure 2*). Case-ascertainment percentages were generally similar across cancer site, stage, and grade of disease though the percentage of AMI captured in both MINAP and HES combined decreased slightly for patients with more tumours, late stage disease or those diagnosed more recently (45% ascertainment of admissions with 4 or more previous tumours, 46% of admissions with a previous stage 4 tumour, and 48% of admissions with a cancer diagnosis in the previous year) (*Figure 3*).

### Mortality after AMI admission

In 95 509 patients followed from their first AMI admission, there were 53 249 deaths over 258 106 person-years of follow-up at a rate of 20.6 [95% confidence interval (CI) 20.5–20.8] per 100 person-years. The crude mortality rate was lower for patients whose AMI admission was captured in both MINAP and HES [16.0 deaths per 100 person-years (95% CI 15.8–16.2)] compared to HES only [31.0 (30.6–31.5)] and MINAP only [22.6 (22.2–23.0)]. Survival differences remained up to 9 years following AMI admission in both STEMI and NSTEMI populations (Supplementary material online, *Figure*  *S2*).

**Figure 4:**
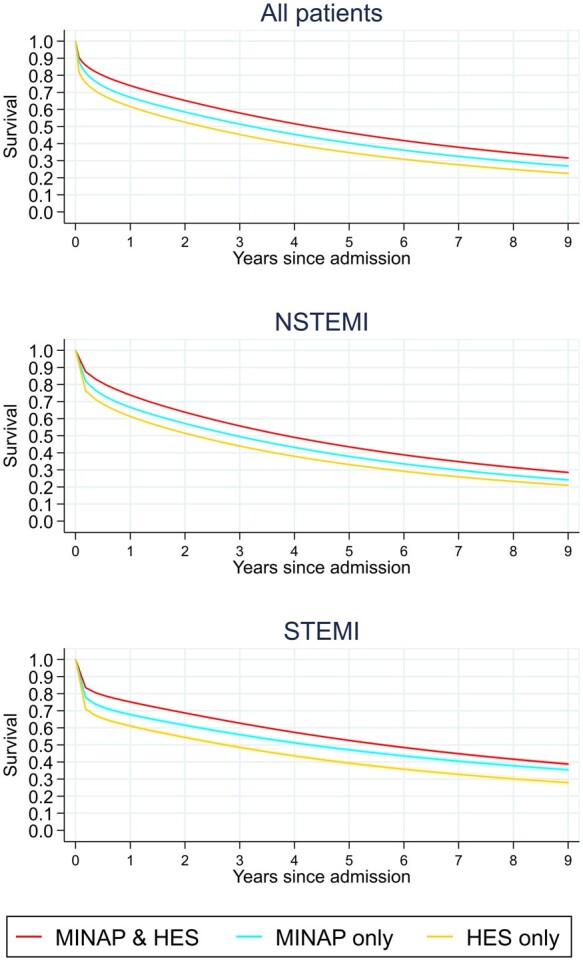
Adjusted survival following hospital admission for myocardial infarction by registry in total (*n* = 95 470) and modelled separately for NSTEMI (*n* = 69 087) and STEMI (*n* = 26 383). Shading indicates 95% confidence interval. Only the first admission for each patient is included. Thirty-one patients were excluded from these analyses as they had unknown AMI phenotype. Analyses were on a complete-case basis. Standardized analyses included: age group (<60, 60–69, 70–79, 80–89, >90 years); sex; ethnicity (White, non-White, unknown); myocardial infarction phenotype (STEMI or NSTEMI); Charlson Comorbidity Index score; index of multiple deprivation quintile; region of CCG (London, Midlands and East, North, South); years between cancer diagnosis and admission (<1 year, 1–5 years, 5–10 years, >10 years); number of previous tumours (1, 2, 3, 4, or more); cancer subtype (invasive or non-invasive/non-melanoma skin cancer); and year of admission. AMI phenotype was not included in the STEMI and NSTEMI models.

Significant differences in survival remained between patients ascertained by the different sources after adjustment. Survival estimates, standardized to the demographics of the full AMI population, were lower for patients whose first AMI admission was captured in HES only, throughout follow-up (*Figure 4*, Supplementary material online, *Table*  *S2*), with 30-day survival of 89.4% (89.2–89.7%) for patients in both MINAP and HES, compared to 80.4% (80.0–80.9%) for HES only patients. By nine years after admission, survival was 31.6% (31.0–32.2%) for patients in both MINAP and HES, compared to 22.6% (21.9–23.3%) for HES only patients. Compared to patients captured in both MINAP and HES, mortality rates were considerably higher in the first year following AMI admission in MINAP only and HES only patients, and continued to remain higher in the NSTEMI population up to 9 years following AMI admission (Supplementary material online, *Figure*  *S3*).

### AMI coding trends

Between 2010 and 2017, coding of AMI changed dramatically in HES with a 33.7% relative increase in AMI admissions coded as ‘Acute MI’ (I21) (6737/9087 in 2010 compared with 12 261/12 374 in 2017), and a 96.7% relative decrease in admissions coded as ‘subsequent MI’ (I22) (2338/9087 in 2010 compared with 105/12 374 in 2017) (Supplementary material online, *Table*  *S3*). I23 codes, indicating ‘complications from MI’, were rarely used across all years. AMI admissions captured in HES only were more likely to have the ‘site unspecified’ (47.4% coded as I21.9) compared to admissions captured by both MINAP and HES (34.8% coded as I21.9) (Supplementary material online, *Table*  *S4*).

### Further examination of MINAP-only admissions

Of 26 104 admissions captured only in MINAP the majority (25 539, 97.8%) could be matched to a HES admission with a non-MI primary diagnosis within 30 days. The most frequent ICD-10 chapters were for diseases of the circulatory system (I00–I99) (13 122, 51.4% of matched admissions) followed by symptoms not elsewhere classified (R00–R99) (5389, 21.1% of matched admissions) (Supplementary material online, *Table*  *S5a*). Specifically, chronic ischaemic heart disease (I25) and angina pectoris (I20) were the most common circulatory-related primary diagnoses, while pain in throat and chest (R07) was the most common symptom-related primary diagnosis (Supplementary material online, *Table*  *S5b*). These accounted for 15.3%, 15.1%, and 14.4% of matched admissions, respectively.

In a separate analysis of 26 104 admissions captured only in MINAP, the majority (15 118, 57.9%) could be matched to a HES admission with an AMI diagnosis in the second diagnosis position or higher and/or the second episode or higher within 30 days (Supplementary material online, *Table*  *S6*).

### Sensitivity analyses

Widening the time window in which admissions were deemed to match between data sources made minimal difference to the case-ascertainment percentages, with admissions captured in both MINAP and HES increasing to 52.1% if a 90-day window either side of the MINAP admission was used (Supplementary material online, *Table*  *S7*). Additionally, broadening the criteria from which AMI admissions were identified from HES resulted in admissions captured by both MINAP and HES increasing to 54.1% if secondary diagnoses in the first episode were also considered, and to 57.3% if AMI captured in any episode of the spell in the primary diagnosis position was considered (Supplementary material online, *Table*  *S8*). Using HES records that captured AMI in any diagnostic position of any episode resulted in a greater number of MINAP records being captured in HES, but a lower case-ascertainment percentage overall given the greater number of HES only cases also identified.

## Discussion

We present the first investigation of case-ascertainment for AMI in a large national observational linked dataset of cardio-oncology patients from VICORI. We describe firstly, a large population of more than 95 000 patients hospitalized with AMI who have a prior cancer diagnosis. Secondly, overlap between MINAP and HES capture of AMI is incomplete and both data sources are needed for a full understanding of hospitalized AMI in a cancer population. Thirdly, episodes ascertained in MINAP only, HES only and in both MINAP and HES identified different types of AMI patients with markedly differing prognoses. Finally, cancer characteristics (site, stage, and grade) had little effect on how AMI was captured. These findings support a strategy of using multiple linked data sources for observational cardio-oncological research into AMI.

We report that, whilst in a cancer population, the overlap between HES and MINAP coding has improved over time, there remain important AMI populations who can be identified from one or another dataset only. Overall, 51% of AMI admissions in cancer patients were captured in both MINAP and HES, a slight improvement on the 46% captured in the same datasets reported by Herrett *et al.*,^8^ who first demonstrated from 2003 to 2009 data that MINAP and HES (and the primary care data source CPRD—Clinical Practice Research Datalink) defined incompletely overlapping AMI populations. We have confirmed that this issue remains pertinent in a more contemporary dataset that is over six times larger, and specifically is of relevance to cancer patients. Furthermore, differences in ascertainment source identified distinct AMI populations. Patients captured in both MINAP and HES were on average the youngest, most likely to be male, have a STEMI presentation, and had the least prevalence of comorbidity, lowest cancer burden, and the lowest 30-day mortality. MINAP only cases were on average older, more likely to be female, more likely to have an NSTEMI presentation, more comorbid and had a higher 30-day mortality whereas cases ascertained in HES only were the oldest, most likely female, most likely to have NSTEMI, most comorbid and had the highest 30-day mortality. Importantly the large differences in survival persisted even after adjustment for patient characteristics.

The likely explanation lies in the different approach and aims of data collection for MINAP and HES. MINAP is a disease-specific audit whose primary aim is to improve the quality of specialist cardiac care for the management of acute coronary syndromes (Type I AMI^9^) Ascertainment in MINAP is therefore at its best for STEMI but will be reduced for AMI cared for exclusively by non-cardiac specialists such as patients being managed palliatively or those who have AMI (particularly NSTEMI) in the context of another, significant diagnosis or comorbidity. It will also be lower for Type II AMI, which is not the primary focus of MINAP. HES is derived from the coding of clinical episodes and aims to capture all comorbidity for England’s National Health Service administrative and funding purposes. HES is less sensitive to hospital speciality but also less specific to refined definitions. It will therefore potentially capture AMI occurring in non-cardiac care hospital locations but also encompass more Type II AMI. It is interesting, for example, to note that the majority of AMI cases ascertained in only MINAP had a corresponding coded HES episode but often with a less specific or non-AMI cardiovascular disease code. It is possible that variations in the permissiveness of coding for AMI, particularly in HES, also account for the regional differences noted in ascertainment between MINAP and HES. It is interesting that patients captured in both MINAP and HES had the lowest risk profile and the lowest mortality. It may be that this group is the simplest to identify as AMI and therefore the least likely to be differentially coded in HES or not included in MINAP.

Taken together these findings strongly support the use of both HES and MINAP data for the ascertainment of AMI in cancer patients. In addition, key cancer characteristics such as site, stage, and grade had a low impact on AMI ascertainment suggesting audit recording and coding of AMI is similar across the cancer population. This implies that valid comparisons can be made between different cancer patients (across the full spectrum of disease) within this linked dataset. These findings suggest this novel linked data resource VICORI can identify a large and representative cardio-oncological cohort enabling robust statistical power coupled with data on a variety of relevant risk factors and comorbidities. Further investigation of cancer treatment cardiotoxicity leading to AMI using the VICORI dataset is necessary.

### Strengths and limitations

This study is subject to a number of limitations. Firstly, we studied only secondary care data sources, and therefore do not have a complete picture of AMI case-ascertainment. Those recorded only in primary care records could make up around one-fifth of non-fatal AMI,^8^ and it has been found that about a half of all fatal AMIs, as recorded on a death certificate, do not have a hospital admission within the preceding 28 days.^14^ Secondly, we could not compare case-ascertainment directly with a non-cancer population. Whilst the percentage of AMI captured in both MINAP and HES of 51% is similar to a previous population-based estimate,^8^ there is no guarantee that these results can be generalized to other diseases, with recent investigations in a population with mild-severe chronic kidney disease or those with known risk factors for kidney dysfunction showing a far lower percentage captured by both data sources (23%, Bidulka *et al.*, manuscript under review). Thirdly, AMI phenotype was determined differently in MINAP and HES. It is possible that this could have resulted in misclassification, particularly in HES as only ICD-10 codes were used to identify phenotype. Finally, the results could be affected by residual confounding. Although we present predominantly descriptive results, we have attempted to understand survival differences by case-ascertainment source through adjustment and standardization. Given that large survival differences remained after adjustment we suspect residual confounding, perhaps via lifestyle, patient frailty, socio-economic factors, geographical differences, and/or as a result of invasive management that occurred during AMI hospital admission (i.e. to treat bleeding and/or coagulopathy). The rates of severe complications and subsequent invasive management of these complications, which are not captured in our data, may have differed depending on AMI phenotype.

This study also has several strengths. The VICORI dataset is a new and unique data resource that includes both detailed patient-level information on cancer pathology and treatment linked with specifics of cardiovascular disease diagnosis and management. Our study included a large, representative cohort of cancer patients who have AMI covering all geographic regions of England allowing for robust statistical power to investigate the outcomes. VICORI also includes linkage with other sources so we were able to obtain and adjust for mortality risk factors including socio-economic deprivation, comorbidities, and ethnicity.

## Conclusions

In patients with a previous diagnosis of cancer, MINAP and HES define different populations of patients with AMI though there is considerable overlap. However, cancer characteristics (site, stage and grade) do not impact on case-ascertainment. Using these data sources together, a large population of cancer patients with AMI can be defined. This provides an important resource for observational population studies and potential clinical trials in cardio-oncology.

## Supplementary material

Supplementary material is available at *European Heart Journal – Quality of Care and Clinical Outcomes* online.

## Supplementary Material

qcab045_Supplementary_Data
