# Supplementary material for: Case-ascertainment of acute myocardial infarction hospitalizations in cancer patients: a cohort study using English linked electronic health data
Source: Eur Heart J Qual Care Clin Outcomes. 2021 Jun 22;8(1):86–95. doi: 10.1093/ehjqcco/qcab045 (PMC8728035; doi:10.1093/ehjqcco/qcab045)
Supplement: qcab045_Supplementary_Data [file qcab045_supplementary_data.docx]

**Case-ascertainment of acute myocardial infarction hospitalisations in cancer patients: a cohort study using English linked electronic health data**

Supplementary Material

Contents

[**Supplementary Table 1.** Case-ascertainment of n=112,502 AMI hospitalisations across disease registry (MINAP) and hospital care (HES) sources for a population of cardio-oncology patients, by sex, AMI phenotype, age, calendar year, ethnicity, and deprivation quintile corresponding to Figure 2. 3](#_Toc66806274)

[**Supplementary Table 2.** Crude and standardised survival across disease registry sources at one-year intervals following first AMI admission. 4](#_Toc66806275)

[**Supplementary Table 3.** In hospital AMI diagnoses ICD-10 codes by AMI admission year (includes for MINAP & HES and HES only admissions). 5](#_Toc66806276)

[**Supplementary Table 4.** In hospital AMI diagnoses ICD-10 codes by disease registry source (includes MINAP & HES and HES only admissions). 7](#_Toc66806277)

[**Supplementary Table 5a.** In hospital non-AMI diagnoses for MINAP only admissions. 8](#_Toc66806278)

[**Supplementary Table 5b.** In hospital non-AMI diagnoses for MINAP only admissions (three-digit ICD-10 codes for I and R). 9](#_Toc66806279)

[**Supplementary Table 6.** In hospital HES AMI diagnoses that were not included in the first diagnostic position of the first episode for admissions coded as “MINAP only”, by diagnosis position and episode number. 10](#_Toc66806280)

[**Supplementary Table 7.** Number and percent of admissions across disease registry sources using different time windows to match AMI hospitalisations. 11](#_Toc66806281)

[**Supplementary Table 8.**  Number and percent of admissions across disease registry sources using different methods to identify AMI hospitalisations in HES. 12](#_Toc66806282)

[**Supplementary Figure 1.** Case-ascertainment of AMI hospitalisations across disease registry sources by sex, age group, and year of admission for STEMI and NSTEMI. 13](#_Toc66806283)

[**Supplementary Figure 2.**  Kaplan Meier survival following hospital admission for myocardial infarction by registry in total (n=95,509) and for NSTEMI (n=69,091) and STEMI (n=26,387). 14](#_Toc66806284)

[**Supplementary Figure 3.**  Standardised mortality hazard ratios by case-ascertainment source. Reference is patients captured in both MINAP & HES. 15](#_Toc66806285)

[**Appendix A.** Criteria for matching patients in the National Cancer Registry and Analysis Service to Hospital Episode Statistics Admitted Patient Care data. 16](#_Toc66806286)

[**Appendix B.** Criteria for identifying ST elevation and non-ST elevation AMI hospitalisations in MINAP 17](#_Toc66806287)

[**Appendix C.** Criteria for determining AMI phenotype (ST elevation and non-ST elevation) in HES 18](#_Toc66806288)

**Supplementary Table 1.** Case-ascertainment of n=112,502 AMI hospitalisations across disease registry (MINAP) and hospital care (HES) sources for a population of cardio-oncology patients, by sex, AMI phenotype, age, calendar year, ethnicity, and deprivation quintile corresponding to Figure 2.

| **Variable** | **Total n** | **MINAP&HES** | **MINAP only** | **HES only** |
| --- | --- | --- | --- | --- |
| **Age group** | **112,502** |  |  |  |
| 40-59 | 6,945 | 60.8% (59.6%-61.9%) | 20.1% (19.1%-21.0%) | 19.2% (18.3%-20.1%) |
| 60-69 | 17,036 | 56.9% (56.2%-57.7%) | 22.0% (21.4%-22.7%) | 21.0% (20.4%-21.6%) |
| 70-79 | 34,899 | 53.1% (52.6%-53.6%) | 23.8% (23.4%-24.3%) | 23.1% (22.6%-23.5%) |
| 80-89 | 41,270 | 47.7% (47.2%-48.2%) | 24.1% (23.6%-24.5%) | 28.2% (27.8%-28.7%) |
| 90+ | 12,352 | 41.4% (40.6%-42.3%) | 22.0% (21.3%-22.8%) | 36.5% (35.7%-37.4%) |
| **Sex** | **112,502** |  |  |  |
| Male | 72,500 | 52.2% (51.8%-52.6%) | 23.3% (22.9%-23.6%) | 24.6% (24.2%-24.9%) |
| Female | 40,002 | 48.6% (48.1%-49.0%) | 23.1% (22.7%-23.5%) | 28.3% (27.9%-28.8%) |
| **Ethnicity** | **85,108** |  |  |  |
| White | 81,831 | 50.9% (50.6%-51.3%) | 23.1% (22.8%-23.4%) | 26.0% (25.7%-26.3%) |
| Mixed | 155 | 43.2% (35.7%-51.1%) | 30.3% (23.6%-38.0%) | 26.5% (20.1%-33.9%) |
| Asian | 1,875 | 46.8% (44.6%-49.1%) | 24.2% (22.3%-26.2%) | 29.0% (27.0%-31.1%) |
| Black | 691 | 39.4% (35.8%-43.1%) | 27.1% (23.9%-30.5%) | 33.6% (30.1%-37.2%) |
| Other | 556 | 47.1% (43.0%-51.3%) | 24.3% (20.9%-28.0%) | 28.6% (25.0%-32.5%) |
| **Pathology** | **112,456** |  |  |  |
| NSTEMI | 83,341 | 45.4% (45.0%-45.7%) | 25.6% (25.3%-25.9%) | 29.1% (28.8%-29.4%) |
| STEMI | 29,115 | 66.9% (66.3%-67.4%) | 16.5% (16.1%-16.9%) | 16.6% (16.2%-17.1%) |
| **Admission year** | **112,502** |  |  |  |
| 2010 | 12,970 | 44.6% (43.8%-45.5%) | 29.9% (29.2%-30.7%) | 25.4% (24.7%-26.2%) |
| 2011 | 13,263 | 44.8% (43.9%-45.6%) | 30.6% (29.8%-31.4%) | 24.6% (23.9%-25.4%) |
| 2012 | 13,842 | 48.6% (47.7%-49.4%) | 24.8% (24.1%-25.5%) | 26.6% (25.9%-27.4%) |
| 2013 | 13,634 | 51.5% (50.7%-52.4%) | 22.1% (21.4%-22.8%) | 26.3% (25.6%-27.1%) |
| 2014 | 14,092 | 53.5% (52.7%-54.3%) | 20.9% (20.3%-21.6%) | 25.6% (24.9%-26.3%) |
| 2015 | 14,576 | 52.5% (51.7%-53.3%) | 20.6% (20.0%-21.3%) | 26.8% (26.1%-27.6%) |
| 2016 | 14,840 | 54.1% (53.3%-54.9%) | 19.2% (18.6%-19.8%) | 26.7% (26.0%-27.4%) |
| 2017 | 15,285 | 56.0% (55.2%-56.8%) | 19.0% (18.4%-19.7%) | 25.0% (24.3%-25.6%) |
| **Comorbidity score** | **112,490** |  |  |  |
| 0 | 18,078 | 54.9% (54.2%-55.7%) | 19.5% (19.0%-20.1%) | 25.5% (24.9%-26.2%) |
| 1 | 9,980 | 53.9% (52.9%-54.8%) | 22.3% (21.5%-23.2%) | 23.8% (23.0%-24.6%) |
| 2 | 28,410 | 53.5% (52.9%-54.0%) | 21.2% (20.7%-21.7%) | 25.3% (24.8%-25.8%) |
| 3 | 19,270 | 50.6% (49.9%-51.4%) | 24.1% (23.5%-24.7%) | 25.2% (24.6%-25.8%) |
| 4 or more | 36,752 | 46.3% (45.8%-46.8%) | 26.3% (25.8%-26.7%) | 27.4% (27.0%-27.9%) |
| **Deprivation quintile** | **112,502** |  |  |  |
| 1 (least deprived) | 22,310 | 51.1% (50.5%-51.8%) | 22.7% (22.1%-23.2%) | 26.2% (25.6%-26.8%) |
| 2 | 24,688 | 51.3% (50.7%-51.9%) | 22.7% (22.2%-23.2%) | 26.0% (25.5%-26.6%) |
| 3 | 24,053 | 50.8% (50.2%-51.5%) | 23.4% (22.8%-23.9%) | 25.8% (25.3%-26.4%) |
| 4 | 21,682 | 50.1% (49.5%-50.8%) | 23.8% (23.3%-24.4%) | 26.0% (25.4%-26.6%) |
| 5 (most deprived) | 19,769 | 51.1% (50.4%-51.8%) | 23.6% (23.0%-24.2%) | 25.4% (24.8%-26.0%) |
| **Region** | **112,502** |  |  |  |
| London | 9,163 | 39.5% (38.5%-40.5%) | 26.3% (25.4%-27.2%) | 34.1% (33.2%-35.1%) |
| Midlands and East | 34,052 | 49.3% (48.8%-49.8%) | 22.5% (22.0%-22.9%) | 28.2% (27.7%-28.7%) |
| North | 38,473 | 55.7% (55.2%-56.2%) | 23.5% (23.0%-23.9%) | 20.8% (20.4%-21.2%) |
| South | 30,814 | 50.1% (49.5%-50.6%) | 22.7% (22.3%-23.2%) | 27.2% (26.7%-27.7%) |

Percentages include percentage (95% confidence interval).

**Supplementary Table 2.** Crude and standardised survival across disease registry sources at one-year intervals following first AMI admission.

| **Years after AMI admission** | **Crude survival** | | | **Standardised survival†** | | |
| --- | --- | --- | --- | --- | --- | --- |
|  | **MINAP & HES** | **MINAP only** | **HES only** | **MINAP & HES** | **MINAP only** | **HES only** |
| **0** | 100.0% (100.0%-100.0%) | 100.0% (100.0%-100.0%) | 100.0% (100.0%-100.0%) | 100.0% (100.0%-100.0%) | 100.0% (100.0%-100.0%) | 100.0% (100.0%-100.0%) |
| **1** | 75.4% (75.0%-75.8%) | 65.3% (64.6%-65.9%) | 57.2% (56.6%-57.8%) | 74.0% (73.6%-74.3%) | 67.1% (66.6%-67.7%) | 61.7% (61.2%-62.2%) |
| **2** | 67.1% (66.7%-67.5%) | 55.8% (55.1%-56.4%) | 47.8% (47.1%-48.4%) | 65.2% (64.8%-65.6%) | 58.5% (57.9%-59.0%) | 52.5% (52.0%-53.1%) |
| **3** | 60.3% (59.9%-60.8%) | 49.0% (48.3%-49.6%) | 40.5% (39.9%-41.1%) | 57.9% (57.5%-58.3%) | 51.4% (50.9%-52.0%) | 45.3% (44.8%-45.9%) |
| **4** | 54.6% (54.1%-55.1%) | 42.9% (42.2%-43.6%) | 34.9% (34.2%-35.5%) | 51.7% (51.3%-52.1%) | 45.4% (44.9%-46.0%) | 39.5% (39.0%-40.0%) |
| **5** | 49.4% (48.9%-49.9%) | 37.8% (37.1%-38.5%) | 30.2% (29.5%-30.8%) | 46.3% (45.9%-46.8%) | 40.4% (39.8%-40.9%) | 34.8% (34.2%-35.3%) |
| **6** | 44.6% (44.0%-45.1%) | 33.5% (32.8%-34.2%) | 26.5% (25.8%-27.2%) | 41.8% (41.3%-42.2%) | 36.1% (35.5%-36.7%) | 30.9% (30.3%-31.5%) |
| **7** | 40.4% (39.8%-41.0%) | 29.8% (29.1%-30.6%) | 23.1% (22.4%-23.8%) | 37.9% (37.4%-38.4%) | 32.5% (31.9%-33.2%) | 27.6% (27.0%-28.3%) |
| **8** | 36.6% (35.9%-37.3%) | 26.7% (25.9%-27.5%) | 20.2% (19.4%-21.1%) | 34.5% (33.9%-35.1%) | 29.5% (28.8%-30.2%) | 24.9% (24.2%-25.6%) |
| **9** | - | - | - | 31.6% (31.0%-32.2%) | 26.9% (26.2%-27.6%) | 22.6% (21.9%-23.3%) |

Crude survival at 30 days was 89.9% (89.6%-90.1%) for MINAP & HES; 85.0% (84.6%-85.5%) for MINAP only; and 77.6% (77.1%-78.1%) for HES only. Standardised survival at 30 days was 89.4% (89.2%-89.7%) for MINAP & HES; 86.7% (86.7%-87.1%) for MINAP only; and 80.4% (80.0%-80.9%) for HES only.

† Standardising across the covariate distribution for all patients in the study.

**Supplementary Table 3.** In hospital AMI diagnoses ICD-10 codes by AMI admission year (includes for MINAP & HES and HES only admissions).

| **ICD-10 code and description** | | **2010** | **2011** | **2012** | **2013** | **2014** | **2015** | **2016** | **2017** | **Total** |
| --- | --- | --- | --- | --- | --- | --- | --- | --- | --- | --- |
| **I21** | **Acute MI** | 6,737 | 6,773 | 9,623 | 10,501 | 11,035 | 11,450 | 11,879 | 12,261 | 80,259 |
| **I21.0** | Acute transmural MI of anterior wall | 790 | 725 | 1,007 | 1,189 | 1,271 | 1,252 | 1,242 | 1,267 | 8,743 |
|  |  | 8.69% | 7.87% | 9.68% | 11.20% | 11.41% | 10.82% | 10.36% | 10.24% | 10.12% |
| **I21.1** | Acute transmural MI of inferior wall | 890 | 883 | 1,260 | 1,419 | 1,455 | 1,521 | 1,611 | 1,616 | 10,655 |
|  |  | 9.79% | 9.59% | 12.11% | 13.36% | 13.06% | 13.15% | 13.44% | 13.06% | 12.33% |
| **I21.2** | Acute transmural MI of other sites | 127 | 130 | 183 | 224 | 215 | 199 | 248 | 247 | 1,573 |
|  |  | 1.40% | 1.41% | 1.76% | 2.11% | 1.93% | 1.72% | 2.07% | 2.00% | 1.82% |
| **I21.3** | Acute transmural MI of unspecified site | 49 | 69 | 62 | 28 | 28 | 486 | 559 | 495 | 1,776 |
|  |  | 0.54% | 0.75% | 0.60% | 0.26% | 0.25% | 4.20% | 4.66% | 4.00% | 2.06% |
| **I21.4** | Acute subendocardial MI | 624 | 669 | 690 | 414 | 281 | 5,475 | 7,559 | 8,090 | 23,802 |
|  |  | 6.87% | 7.26% | 6.63% | 3.90% | 2.52% | 47.32% | 63.04% | 65.38% | 27.55% |
| **I21.9** | Acute MI, unspecified | 4,257 | 4,297 | 6,421 | 7,227 | 7,785 | 2,517 | 660 | 546 | 33,710 |
|  |  | 46.85% | 46.66% | 61.70% | 68.06% | 69.86% | 21.76% | 5.50% | 4.41% | 39.02% |
| **I22** | **Subsequent MI** | 2,338 | 2,430 | 776 | 111 | 102 | 110 | 110 | 105 | 6,082 |
| **I22.0** | Subsequent MI of anterior wall | 137 | 124 | 45 | 15 | 6 | 11 | 6 | <5 | 347 |
|  |  | 1.51% | 1.35% | 0.43% | 0.14% | 0.05% | 0.10% | 0.05% | 0.02% | 0.40% |
| **I22.1** | Subsequent MI of inferior wall | 150 | 149 | 57 | 9 | 18 | 11 | 7 | 10 | 411 |
|  |  | 1.65% | 1.62% | 0.55% | 0.08% | 0.16% | 0.10% | 0.06% | 0.08% | 0.48% |
| **I22.8** | Subsequent MI of other sites | 197 | 201 | 56 | <5 | <5 | 20 | 18 | 17 | 517 |
|  |  | 2.17% | 2.18% | 0.54% | 0.04% | 0.04% | 0.17% | 0.15% | 0.14% | 0.60% |
| **I22.9** | Subsequent MI of unspecified site | 1,854 | 1,956 | 618 | 83 | 74 | 68 | 79 | 75 | 4,807 |
|  |  | 20.4% | 21.24% | 5.94% | 0.78% | 0.66% | 0.59% | 0.66% | 0.61% | 5.56% |
| **I23** | **Certain current complications following acute MI** | 12 | 6 | 7 | 7 | 7 | 9 | <5 | 8 | 57 |
| **I23.0** | Haemopericardium as current complication following acute MI | 0 | 0 | <5 | <5 | 0 | 0 | 0 | <5 | <5 |
|  |  | - | - | 0.01% | 0.02% | - | - | - | 0.01% | 0.00% |
| **I23.1** | Atrial septal defect as current complication following acute MI | 0 | <5 | <5 | 0 | <5 | <5 | 0 | 0 | 7 |
|  |  | - | 0.01% | 0.03% | - | 0.01% | 0.02% | - | - | 0.01% |
| **I23.2** | Ventricular septal defect as current complication following acute MI | 5 | <5 | <5 | <5 | <5 | 5 | <5 | <5 | 22 |
|  |  | 0.06% | 0.01% | 0.01% | 0.04% | 0.03% | 0.04% | 0.01% | 0.02% | 0.03 |
| **I23.3** | Rupture of cardiac wall without haemopericardium as current complication following acute MI | <5 | 0 | <5 | 0 | 0 | <5 | 0 | 0 | <5 |
|  |  | 0.01% | 0 | 0.01% | - | - | 0.01% | - | - | 0.00% |
| **I23.4** | Rupture of chordae tendineae as current complication following acute MI | 0 | 0 | 0 | <5 | 0 | 0 | 0 | 0 | <5 |
|  |  | - | - | - | 0.01% | - | - | - | - | 0.00% |
| **I23.6** | Thrombosis of atrium, auricular appendage, and ventricle as current complications following acute MI | <5 | <5 | 0 | 0 | <5 | <5 | 0 | <5 | 9 |
|  |  | 0.02% | 0.02% | - | - | 0.01% | 0.01% | - | 0.02% | 0.01% |
| **I23.8** | Other current complications following acute MI | <5 | <5 | <5 | 0 | <5 | 0 | 0 | <5 | 11 |
|  |  | 0.04% | 0.02% | 0.01% | - | 0.02% | - | - | 0.02% | 0.01% |
| **Total** | | **9,087** | **9,209** | **10,406** | **10,619** | **11,144** | **11,569** | **11,990** | **12,374** | **86,398** |
|  |  | **100.00%** | **100.00%** | **100.00%** | **100.00%** | **100.00%** | **100.00%** | **100.00%** | **100.00%** | **100.00%** |

I23.5 (Rupture of papillary muscle as current complication following acute myocardial infarction) is not included in the table as there were no admissions with this code.

**Supplementary Table 4.** In hospital AMI diagnoses ICD-10 codes by disease registry source (includes MINAP & HES and HES only admissions).

| **ICD-10 and description** | **Case-ascertainment source** | | **Total** |
| --- | --- | --- | --- |
|  | **MINAP & HES** | **HES only** |  |
| I21 Acute MI | 53,398 | 26,861 | 80,259 |
| I21.0 Acute transmural MI of anterior wall | 7,009 | 1,734 | 8,743 |
|  | 12.2% | 6.0% | 10.1% |
| I21.1 Acute transmural MI of inferior wall | 8,911 | 1,744 | 10,655 |
|  | 15.6% | 6.0% | 12.3% |
| I21.2 Acute transmural MI of other sites | 1,162 | 411 | 1,573 |
|  | 2.0% | 1.4% | 1.8% |
| I21.3 Acute transmural MI of unspecified site | 1,211 | 565 | 1,776 |
|  | 2.1% | 1.9% | 2.1% |
| I21.4 Acute subendocardial MI | 15,204 | 8,598 | 23,802 |
|  | 26.6% | 29.5% | 27.6% |
| I21.9 Acute MI, unspecified | 19,901 | 13,809 | 33,710 |
|  | 34.8% | 47.4% | 39.0% |
| I22 Subsequent MI | 3,856 | 2,226 | 6,082 |
| I22.0 Subsequent MI of anterior wall | 264 | 83 | 347 |
|  | 0.5% | 0.3% | 0.4% |
| I22.1 Subsequent MI of inferior wall | 327 | 84 | 411 |
|  | 0.6% | 0.3% | 0.5% |
| I22.8 Subsequent MI of other sites | 299 | 218 | 517 |
|  | 0.5% | 0.8% | 0.6% |
| I22.9 Subsequent MI of unspecified site | 2,966 | 1,841 | 4,807 |
|  | 5.2% | 6.3% | 5.6% |
| I23 Certain current complications following acute MI | 11 | 46 | 57 |
| I23.0 Haemopericardium as current complication following acute MI | 0 | 4 | 4 |
|  | - | 0.0% | 0.0% |
| I23.1 Atrial septal defect as current complication following acute MI | 0 | 7 | 7 |
|  | - | 0.0% | 0.0% |
| I23.2 Ventricular septal defect as current complication following acute MI | 6 | 16 | 22 |
|  | 0.0% | 0.1% | 0.0% |
| I23.3 Rupture of cardiac wall without haemopericardium as current complication following acute MI | 1 | 2 | 3 |
|  | 0.0% | 0.0% | 0.0% |
| I23.4 Rupture of chordae tendineae as current complication following acute MI | 0 | 1 | 1 |
|  | - | 0.0% | 0.0% |
| I23.6 Thrombosis of atrium, auricular appendage, and ventricle as current complications following acute MI | 1 | 8 | 9 |
|  | 0.0% | 0.0% | 0.0% |
| I23.8 Other current complications following acute MI | 3 | 8 | 11 |
|  | 0.0% | 0.0% | 0.0% |
| **Total admissions** | 57,265 | 29,133 | 86,398 |
|  | 100.0% | 100.0% | 100.0% |

I23.5 (Rupture of papillary muscle as current complication following acute myocardial infarction) is not included in the table as there were no admissions with this code.

**Supplementary Table 5a.** In hospital non-AMI diagnoses for MINAP only admissions.

| **ICD-10** | | **Description** | **n** | **%** |
| --- | --- | --- | --- | --- |
| A | A00-B99 | Certain infectious and parasitic diseases | 365 | 1.43% |
| B |  |  | 8 | 0.03% |
| C | C00-C97 | Malignant neoplasms | 1,097 | 4.30% |
| D | D00-D48 | Other neoplasms | 59 | 0.23% |
|  | D50-D89 | Diseases of the blood and blood-forming organs and certain disorders involving the immune mechanism | 121 | 0.47% |
| E | E00-E89 | Endocrine, nutritional and metabolic diseases | 196 | 0.77% |
| F | F01-F99 | Mental, Behavioural and Neurodevelopmental disorders | 40 | 0.16% |
| G | G00-G99 | Diseases of the nervous system | 114 | 0.45% |
| H | H00-H59 | Diseases of the eye and adnexa | 18 | 0.07% |
|  | H60-H95 | Diseases of the ear and mastoid process | 10 | 0.04% |
| I | I00-I99 | Diseases of the circulatory system | 13,122 | 51.38% |
| J | J00-J99 | Diseases of the respiratory system | 2,263 | 8.86% |
| K | K00-K95 | Diseases of the digestive system | 625 | 2.45% |
| L | L00-L99 | Diseases of the skin and subcutaneous tissue | 101 | 0.40% |
| M | M00-M99 | Diseases of the musculoskeletal system and connective tissue | 321 | 1.26% |
| N | N00-N99 | Diseases of the genitourinary system | 636 | 2.49% |
| O | O00-O99 | Pregnancy, childbirth and the puerperium | <5 | 0.00% |
| P | P00-P96 | Certain conditions originating in the perinatal period | 0 | - |
| Q | Q00-Q99 | Congenital malformations, deformations and chromosomal abnormalities | 5 | 0.02% |
| R | R00-R99 | Symptoms, signs and abnormal clinical and laboratory findings, not elsewhere classified | 5,389 | 21.10% |
| S | S00-T88 | Injury, poisoning and certain other consequences of external causes | 812 | 3.18% |
| T |  |  | 204 | 0.80% |
| V | V00-Y99 | External causes of morbidity | 0 | - |
| Y |  |  | 0 | - |
| Z | Z00-Z99 | Factors influencing health status and contact with health services | 32 | 0.13% |
| **Total admissions** | | | **25,539** | **100.00%** |

**Supplementary Table 5b.** In hospital non-AMI diagnoses for MINAP only admissions (three-digit ICD-10 codes for I and R).

| **ICD-10 and description** | | | **n** | |
| --- | --- | --- | --- | --- |
| I00-I99 Diseases of the circulatory system | | | 13,122 |  |
|  | I00-I02 Acute rheumatic fever | | 0 |  |
|  | I05-I09 Chronic rheumatic heart diseases | | 17 |  |
|  | I10-I15 Hypertensive diseases | | 35 |  |
|  | I20-I25 Ischaemic heart diseases | | 9,863 |  |
|  |  | I20 Angina pectoris | | 3,864 |
|  |  | I24 Other acute ischaemic heart diseases | | 2,104 |
|  |  | I25 Chronic ischaemic heart disease | | 3,895 |
|  | I26-I28 Pulmonary heart disease and diseases of pulmonary circulation | | 46 |  |
|  | I30-I52 Other forms of heart disease | | 2,602 |  |
|  | I60-I69 Cerebrovascular diseases | | 302 |  |
|  | I70-I79 Diseases of arteries, arterioles and capillaries | | 182 |  |
|  | I80-I89 Diseases of veins, lymphatic vessels and lymph nodes, not elsewhere classified | | 19 |  |
|  | I95-I99 Other and unspecified disorders of the circulatory system | | 56 |  |
| R00-R99 Symptoms, signs and abnormal clinical and laboratory findings, not elsewhere classified | | | 5,389 |  |
|  | R00-R09 Symptoms and signs involving the circulatory and respiratory systems | | 4,158 |  |
|  |  | R00 Abnormalities of heart beat | | 63 |
|  |  | R01 Cardiac murmurs and other cardiac sounds | | 0 |
|  |  | R02 Gangrene, not elsewhere classified | | <5 |
|  |  | R03 Abnormal blood-pressure reading, without diagnosis | | <5 |
|  |  | R04 Haemorrhage from respiratory passages | | 18 |
|  |  | R05 Cough | | 8 |
|  |  | R06 Abnormalities of breathing | | 378 |
|  |  | R07 Pain in throat and chest | | 3,680 |
|  |  | R09 Other symptoms and signs involving the circulatory and respiratory systems | | 6 |
|  | R10-R19 Symptoms and signs involving the digestive system and abdomen | | 195 |  |
|  | R20-R23 Symptoms and signs involving the skin and subcutaneous tissue | | 7 |  |
|  | R25-R29 Symptoms and signs involving the nervous and musculoskeletal systems | | 116 |  |
|  | R30-R39 Symptoms and signs involving the urinary system | | 88 |  |
|  | R40-R46 Symptoms and signs involving cognition, perception, emotional state and behaviour | | 112 |  |
|  | R47-R49 Symptoms and signs involving speech and voice | | 8 |  |
|  | R50-R69 General symptoms and signs | | 681 |  |
|  | R70-R79 Abnormal findings on examination of blood, without diagnosis | | 8 |  |
|  | R80-R82 Abnormal findings on examination of urine, without diagnosis | | 0 |  |
|  | R83-R89 Abnormal findings on examination of other body fluids, substances and tissues, without diagnosis | | 0 |  |
|  | R90-R94 Abnormal findings on diagnostic imaging and in function studies, without diagnosis | | 16 |  |
|  | R95-R99 Ill-defined and unknown causes of mortality | | 0 |  |
| **Total I and R admissions** | | | **18,511** |  |

Note, I21-I23 are excluded as these admissions were included in MINAP & HES.

**Supplementary Table 6.** In hospital HES AMI diagnoses that were not included in the first diagnostic position of the first episode for admissions coded as “MINAP only”, by diagnosis position and episode number.

| **Diagnosis position** | **Episode number** | | | | | **Total** |
| --- | --- | --- | --- | --- | --- | --- |
|  | **1** | **2** | **3** | **4** | **5 or higher** |  |
| **1** | 0* | 6,222 | 1,400 | 363 | 174 | **8,159** |
|  | - | 84.1% | 78.3% | 75.6% | 71.6% | **54.0%** |
| **2** | 3,741 | 683 | 200 | 49 | 29 | **4,702** |
|  | 71.8% | 9.2% | 11.2% | 10.2% | 11.9% | **31.1%** |
| **3** | 837 | 241 | 93 | 26 | 17 | **1,214** |
|  | 16.1% | 3.3% | 5.2% | 5.4% | 7.0% | **8.0%** |
| **4** | 328 | 120 | 41 | 13 | 12 | **514** |
|  | 6.3% | 1.6% | 2.3% | 2.7% | 4.9% | **3.4%** |
| **5 or higher** | 305 | 131 | 53 | 29 | 11 | **529** |
|  | 5.9% | 1.8% | 3.0% | 6.0% | 4.5% | **3.5%** |
| **Total** | **5,211** | **7,397** | **1,787** | **480** | **243** | **15,118** |
|  | **100.0%** | **100.0%** | **100.0%** | **100.0%** | **100.0%** | **100.0%** |

*AMI hospitalisation identified in HES from I21-I23 in diagnosis position 1 for first episode in spell were not included in this table as these would be classified as both “MINAP and HES” admissions in the main analysis.

**Supplementary Table 7.** Number and percent of admissions across disease registry sources using different time windows to match AMI hospitalisations.

| **Case-ascertainment source** | **(+/- 30 days)^1^** | | **+/- 60 days** | | **+/- 90 days** | | **No time restriction** | |
| --- | --- | --- | --- | --- | --- | --- | --- | --- |
|  | **n** | **%** | **n** | **%** | **n** | **%** | **n** | **%** |
| MINAP & HES | 57,265 | 50.9% | 57,824 | 51.7% | 58,140 | 52.1% | 61,036 | 56.1% |
| MINAP only | 26,104 | 23.2% | 25,545 | 22.8% | 25,229 | 22.6% | 22,333 | 20.5% |
| HES only | 29,133 | 25.9% | 28,502 | 25.5% | 28,170 | 25.3% | 25,511 | 23.4% |
| **Total admissions** | **112,502** | **100.0%** | **111,871** | **100.0%** | **111,539** | **100.0%** | **108,880** | **100.0%** |

^1^Main analysis.

**Supplementary Table 8.**  Number and percent of admissions across disease registry sources using different methods to identify AMI hospitalisations in HES.

| **Case-ascertainment source** | **Primary diagnosis, first episode^1^** | | **Primary or secondary diagnosis, first episode^2^** | | **Primary diagnosis, any episode^3^** | | **Any diagnosis, any episode^4^** | |
| --- | --- | --- | --- | --- | --- | --- | --- | --- |
|  | **n** | **%** | **n** | **%** | **n** | **%** | **n** | **%** |
| MINAP & HES | 57,265 | 50.9% | 61,926 | 54.1% | 65,402 | 57.3% | 72,345 | 46.3% |
| MINAP only | 26,104 | 23.2% | 21,443 | 18.7% | 17,967 | 15.8% | 11,024 | 7.1% |
| HES only | 29,133 | 25.9% | 31,211 | 27.2% | 30,721 | 26.9% | 72,769 | 46.6% |
| **Total admissions** | **112,502** | **100.0%** | **114,580** | **100.0%** | **114,090** | **100.0%** | **156,138** | **100.0%** |

*^1^Main analysis- AMI hospitalisation identified in HES from I21-I23 in diagnosis position 1 for first episode in spell*

*^2^AMI hospitalisation identified in HES from I21-I23 in diagnosis position 1 or 2 for first episode in spell*

*^3^AMI hospitalisation identified in HES from I21-I23 in diagnosis position 1 for any episode in spell*

*^4^AMI hospitalisation identified in HES from I21-I23 in any diagnosis position for any episode in spell*

**Supplementary Figure 1.** Case-ascertainment of AMI hospitalisations across disease registry sources by sex, age group, and year of admission for STEMI and NSTEMI.

Admission years are 2010-2017.

**Supplementary Figure 2.**  Kaplan Meier survival following hospital admission for myocardial infarction by registry in total (n=95,509) and for NSTEMI (n=69,091) and STEMI (n=26,387).

Only the first admission for each patient is included. Shading indicates 95% confidence interval.

**Supplementary Figure 3.**  Mortality hazard ratios by case-ascertainment source. Reference is patients captured in both MINAP & HES.

**Appendix A.** Criteria for matching patients in the National Cancer Registry and Analysis Service to Hospital Episode Statistics Admitted Patient Care data.

| **Match Rank** | **NHS Number** | **Date of Birth** | **Sex** | **Postcode** |
| --- | --- | --- | --- | --- |
| 1A | Exact | Exact | Exact | Exact |
| 1B | Exact | Exact |  | Exact |
| 2 | Exact | Exact | Exact |  |
| 3 | Exact | Partial | Exact | Exact |
| 4 | Exact | Partial | Exact |  |
| 5 | Exact |  |  | Exact |

Adapted from <https://digital.nhs.uk/data-and-information/publications/statistical/hes-did-data-linkage-report>

**Appendix B.** Criteria for identifying ST elevation and non-ST elevation AMI hospitalisations in MINAP

| **AMI Phenotype** | **Discharge Diagnosis** | **Enzymes Elevated** | **ECG determining treatment** |
| --- | --- | --- | --- |
| **STEMI** | 1. Myocardial infarction (ST elevation) | 1. Yes  NULL. Missing | 1. ST segment elevation  2. Left bundle branch block  NULL. Missing |
|  |  | 0. No^1^ | 1. ST segment elevation^1^ |
|  | 2. Myocardial infarction (non ST elevation)^2^  4. Acute coronary syndrome (troponin positive)/ nSTEMI | 1. Yes  NULL. Missing | 1. ST segment elevation |
|  | 5. Acute coronary syndrome (troponin negative)  10. Acute coronary syndrome (troponin unknown) | 1. Yes | 1. ST segment elevation |
| **NSTEMI** | 1. Myocardial infarction (ST elevation) | 1. Yes  NULL. Missing | 0. No acute changes  3. ST segment depression  4. T wave changes only  5. Other acute abnormality  6. Normal ECG |
|  | 2. Myocardial infarction (non ST elevation)^2^  4. Acute coronary syndrome (troponin positive)/ nSTEMI | 1. Yes  NULL. Missing | 0. No acute changes  2. Left bundle branch block  3. ST segment depression  4. T wave changes only  5. Other acute abnormality  6. Normal ECG  NULL. Missing |
|  | 5. Acute coronary syndrome (troponin negative)  10. Acute coronary syndrome (troponin unknown) | 1. Yes | 0. No acute changes  2. Left bundle branch block  3. ST segment depression  4. T wave changes only  5. Other acute abnormality  6. Normal ECG  NULL. Missing |
| ^1^This combination is coded as “Not Acute MI” in CALIBER.  ^2^This discharge diagnosis code was not present in MINAP for the CALIBER study. | | | |

Modified from criteria used in the CALIBER programme, see <https://www.caliberresearch.org/portal>.

**Appendix C.** Criteria for determining AMI phenotype (ST elevation and non-ST elevation) in HES

| **AMI Phenotype** | **ICD-10 Code** |
| --- | --- |
| **STEMI** | **I21.0:** Acute myocardial infarction of anterior wall  **I21.1:** Acute myocardial infarction of inferior wall  **I21.2:** Acute myocardial infarction of other sites  **I21.3:** Acute myocardial infarction of unspecified sites  **I22.0:** Subsequent myocardial infarction of anterior wall  **I22.1:** Subsequent myocardial infarction of inferior wall  **I22.8:** Subsequent myocardial infarction of other sites |
| **NSTEMI** | **I21.4:** Acute subendocardial myocardial infarction  **I21.9:** Acute myocardial infarction, unspecified  **I22.9:** Subsequent myocardial infarction of unspecified site |
| **Unknown** | **I23.1:** Atrial septal defect as current complication following AMI  **I23.2:** Ventricular septal defect as current complication following AMI  **I23.3:** Rupture of cardiac wall without haemopericardium as complication following AMI  **I23.4:** Rupture of chordae tendineae as complication following AMI  **I23.5:** Rupture of papillary muscle as complication following AMI  **I23.6:** Thrombosis of atrium, auricular appendage, and ventricle as complications following AMI  **I23.8:** Other complications following AMI |

Adapted from UK Biobank phenotype code list (<https://biobank.ndph.ox.ac.uk/showcase/showcase/docs/alg_outcome_mi.pdf>).
